# Adeno-Associated Virus Toxicity in Duchenne Muscular Dystrophy: Mechanisms and Clinical Considerations

**DOI:** 10.3390/genes17030284

**Published:** 2026-02-27

**Authors:** Ezgi Saylam, Eleonora S. D’ambrosio, Maria Tozzo Pesco, Liubov V. Gushchina

**Affiliations:** 1 The Jerry R. Mendell Center for Gene Therapy, Nationwide Children’s Hospital, Columbus, OH 43215, USA; ezgi.saylam@nationwidechildrens.org; 2 Departments of Neurology, UMass Chan Medical School, Worcester, MA 01655, USA; eleonora.dambrosio@umassmed.edu; 3 Departments of Genetic and Cellular Medicine, UMass Chan Medical School, Worcester, MA 01655, USA; maria.tozzopesco@umassmed.edu; 4 Department of Pediatrics, The Ohio State University, Columbus, OH 43215, USA

**Keywords:** Duchenne muscular dystrophy, *DMD* gene, adeno-associated virus, AAV toxicity

## Abstract

**Background/Objectives**: Recombinant adeno-associated virus (AAV) vectors have revolutionized gene therapy for monogenic diseases such as Duchenne muscular dystrophy (DMD). However, high systemic doses required for muscle transduction cause a spectrum of toxicities ranging from transient hepatic inflammation to fatal multi-organ failure leading to death. These adverse events have reshaped the risk–benefit considerations for gene therapy in DMD. **Methods**: We conducted a narrative review describing complications associated with AAV-mediated gene therapies in the DMD field. PubMed and Clinicaltrials databases were used to search for peer-reviewed manuscripts published between 1987 and 2025. Publicly available abstracts and press releases were also used to describe AAV-mediated adverse events that have been discovered. Priority was given to large prospective cohorts, meta-analyses, and high-impact publications. **Results**: We outlined the mechanistic basis of AAV toxicity—spanning innate and adaptive immune activation, vector–host interactions, transgene overexpression, and host vulnerability—and discussed their therapeutic implications for DMD. We also highlighted ongoing strategies for vector re-design, immune modulation, patient selection, and regulatory adaptation, aiming to improve efficacy with safety in the next generation of muscular dystrophy gene therapies. **Conclusions**: Patient safety remains the number one priority in the AAV-mediated gene therapies field. Achieving long-term benefits requires continued optimization of existing vectors, implementation of strict criteria for patient selection, and regulation of immune responses, with close collaboration and transparent dialog among scientists, clinicians, and regulatory agencies, informed by both successful cases as well as tragic deaths reported in the fields of neuromuscular diseases.

## 1. Introduction

Duchenne muscular dystrophy (DMD) is an X-linked neuromuscular disease that affects approximately 1 in 5200 newborn males [1,2] and is caused by mutations in the *DMD* gene, leading to a lack of dystrophin in skeletal muscles and the heart. Loss of dystrophin disrupts sarcolemmal integrity, leading to increased membrane fragility and susceptibility to contraction-induced injury. This instability results in progressive myofiber damage and myonecrosis involving the skeletal muscle, diaphragm, and cardiac muscle, ultimately causing a loss of ambulation, progressive respiratory insufficiency, dilated cardiomyopathy, and premature death. For the last four decades, following the isolation of the *DMD* gene [3], many different gene therapy approaches have been developed to treat DMD, despite numerous challenges, including the very large size of the wild-type *DMD* gene, high rate of spontaneous mutations, immunogenicity to dystrophin, AAV vectors, and the transgene.

Adeno-associated virus (AAV)-mediated micro-dystrophin replacement has become the most advanced gene therapy method for DMD. The recombinant AAV (rAAV) platform, which has low pathogenicity, limited immunogenicity, and natural muscle tropism, has made it possible to deliver truncated dystrophin cDNAs capable of partially restoring dystrophin function. Here, we also summarize micro-dystrophin constructs and then focus on AAV-related toxicities in DMD and clinical implications.

The central concept of the micro-dystrophin approach is that expression of a shortened but functional dystrophin protein can shift the disease phenotype from Duchenne to a Becker-like muscular dystrophy. This strategy is grounded in clinical observations that certain large in-frame deletions of the *DMD* gene—removing up to 46% of the coding sequence—still permit production of partially functional dystrophin, allowing affected individuals to remain ambulant into their sixth decade of life [4].

The FDA-approved first gene replacement therapy for DMD, Elevidys, also known as delandistrogene moxeparvovec-rokl, received an accelerated approval for boys 4–5 years old, based on dystrophin levels on muscle biopsy as a surrogate biomarker in 2023. This was followed by full approval for boys aged 4 and above in 2024 [5]. Following the success of and lessons learned from the Elevidys gene therapy experience, multiple companies have pursued micro-dystrophin gene therapy trials using different AAV vectors and incorporating distinct dystrophin domains into their constructs.

Elevidys uses the micro-dystrophin construct that consists of a codon-optimized micro-dystrophin transgene lacking spectrin-like repeat domains R4-R23 and the CT domain. This specific micro-dystrophin construct (ΔR4-R23/ΔCT) was designed to fit within AAV’s limited packaging capacity while preserving key functional domains: the N-terminus for f-actin binding, spectrin repeats 1–3 and 24, hinges 1, 2, and 4, and the cysteine-rich domain for β-dystroglycan binding [6]. Transgene expression is driven by the muscle-specific MHCK7 promoter to achieve robust expression in both cardiac and skeletal muscle, while using the muscle-tropic AAVrh74 vector. Although Elevidys had raised high hopes in the neuromuscular community, its functional outcome measures, North Star Ambulatory Assessment (NSAA), did not show statistically significant differences at week 52. Secondary functional endpoints favored treatment, which was the justification for full approval, although there was no statistical difference [7]. In March and June 2025, Sarepta reported that two non-ambulatory DMD patients treated with delandistrogene moxeparvovec-rokl had died of acute liver failure, which was attributed to the drug-related complication. As a result, Sarepta suspended shipment of delandistrogene moxeparvovec-rokl to non-ambulatory individuals in July 2025.

There are five other micro-dystrophin drugs in clinical trials sponsored by Solid Biosciences, Genethon, Regenxbio, Insmed, and Pfizer. These therapies differ in terms of promoters, AAV serotypes, hinge, and dystrophin domain inclusion.

Pfizer’s fordadistrogene movaparvovec, PF-06939926 (USA), is an AAV9 vector encoding a codon-optimized ΔR3-R21ΔCT construct under control of a muscle-specific synthetic promoter (hybrid creatine kinase). Preclinical studies showed that this vector produces a miniaturized but functional form of dystrophin, therefore called “mini-dystrophin”. There was no clinical difference in the phase-3 clinical trial (NCT04281485), despite high levels of micro-dystrophin expression (85%) [8]. There were two patient deaths reported in clinical trials following fordadistrogene movaparvovec administration (NCT03362502, NCT05429372). Along with other serious side effects, including thrombocytopenia, acute kidney injury attributed to complement activation in ambulatory individuals occurred; Pfizer ultimately discontinued the program for both efficacy and safety reasons [9].

Solid Biosciences has also developed the micro-dystrophin construct, SGT-001, which underwent phase 1/2 evaluation in ambulatory and non-ambulatory patients (NCT03368742). The program had two temporary clinical holds in 2018 and 2019 due to serious events related to complement activation. Design changes were implemented to address safety concerns, and currently, SGT-003 is in early- to mid-phase evaluation (NCT06138639).

Genethon’s GNT-0004 uses a recombinant AAV2/8 vector delivering a micro-dystrophin transgene, which is currently in clinical trials. Preclinical studies showed normalization of creatine kinase (CK) levels, improved skeletal muscle and cardiac function in DMDmdx animals treated with GNT-0004. However, unexpected cardiac arrhythmias leading to cardiac deaths have been observed with supra-optimal doses, highlighting the need for close cardiac monitoring [10].

Regenxbio’s RGX-202 (USA) is an AAV8-based micro-dystrophin gene therapy that is currently in clinical trials (NCT05693142). The construct is designed to retain the dystrophin C-terminal domain, with the goal of preserving interactions within the dystrophin-associated protein complex while remaining compatible with AAV vector packaging constraints. Insmed’s INS1201 is also in clinical trials and recently started recruiting ambulatory patients to study the safety and biodistribution of the product (NCT06817382). Unlike other micro-dystrophin products that are administered intravenously, INS1201 is delivered through the intrathecal route, representing a different delivery method which may lead to distinct bioavailability.

## 2. Overview of AAV Vectors in DMD

Adeno-associated virus (AAV) is a small, non-enveloped, single-stranded DNA virus from the Parvoviridae family. First identified in the 1960s as a “contaminant” in adenovirus preparations, AAV has since become the vector of choice for in vivo gene therapy. AAV is considered non-pathogenic in humans, and its replication requires the presence of a helper virus. The AAV genome includes 145 base pair (bp) palindromic sequences known as inverted terminal repeats (ITRs), playing critical roles in replication and genome packaging. The AAV genome also contains two major open reading frames (ORFs), encoding REP proteins (Rep78, Rep68, Rep52, Rep40) and CAP proteins (VP1, VP2, VP3), controlled by different promoters: p5, p19, p40, and p81 [11]. The recombinant AAV genome is derived from the wild-type AAV by removing the rep and cap genes, resulting in the deletion of approximately 96% of the native viral genome. These regions are replaced with a therapeutic expression cassette, typically consisting of a promoter, the transgene, and polyadenylation sequences. ITRs are the only AAV-derived sequences retained in the rAAV vectors as they are essential for genome packaging, replication, and episomal stability within host cells after transduction [12]. Removal of the rep and cap ORFs, rendering rAAV vectors replication-deficient, improves safety by preventing uncontrolled viral replication. rAAV gene therapy consists of two key components: the AAV capsid and the recombinant AAV genome. Different AAV serotypes have distinct tissue tropism based on their capsid structures and receptor binding mechanisms. At least 13 natural AAV serotypes and over 100 variants have been identified to date [13,14]. AAV9, AAV8, and AAV7 serotypes exhibit strong tropisms for liver cells, with AAV9 showing the highest number of copies detected in the skeletal and cardiac muscles and rapid-onset kinetics following systemic delivery in animals [14]. Notably, AAV9 is known for its ability to cross the blood–brain barrier, and it is the most favored serotype for neurological and neuromuscular disorders [15], achieving more efficient cell transduction than other serotypes. As a result, the AAV9 capsid was selected for onasemnogene abeparvovec (Zolgensma), the first FDA-approved gene therapy to treat patients with spinal muscular atrophy (SMA).

The vectors used by different companies vary for DMD. Sarepta Therapeutics uses a rhesus monkey-derived AAV rh74 serotype (AAVrh74), Solid Biosciences and Pfizer use AAV9, and Genethon, with Regenxbio preferring the AAV8 serotype (Table 1).

Currently, there are more than 340 AAV gene therapy clinical trials worldwide, and muscular dystrophies, especially DMD, are among the most actively studied diseases [11,12]. Developments in research have revealed numerous challenges in the gene therapy era. Immunogenicity remains the most significant safety concern. Treating the entire muscle system requires very high systemic doses of AAV vectors, which have been linked to serious dose-related side effects [16,17]. In addition, the small AAV packaging capacity (~4.7 kb) limits the delivery of full-length dystrophin (~14 kb). Vector dosing is weight-dependent, such that older/ heavier patients require substantially higher doses, further increasing the risk of toxicity. Timing of AAV-based therapy is another challenge: early administration may result in dilution of transgene expression due to ongoing muscle regeneration, whereas treatment at later disease stages may be less effective because dystrophic muscle pathology impairs AAV transduction efficiency [5,18,19].

**Table 1:** The structural design of the micro-dystrophin in clinical trials ^1^*.

AAV Serotype	Promoter	Transgene	Name	Comment
AAVrh74	MHCK7	ΔR4-23, ΔCT	Elevidys/Delandistrogene moxeparvovec-rokl	Sarepta
AAV8	Spc5-12	ΔR4-23, ΔCT	GNT00004	Genethon
AAV8	Spc5-12	ΔH2-R19, ΔR20-23, partial CT	RGX-202	Regenxbio
AAV9/SLB-101	CK8e	ΔSR2, ΔSR18-22, ΔCT	SGT-003	Solid Bio
AAV9	CK7	Δr3-19, ΔR20-21, ΔCT	PF-06939926/Fordadistrogene movaparvovec	Pfizer (discontinued)
AAV9	MHCK7	Contains exons 1–17 and 59–69	n/a	Insmed (transgene design not published)

^1^* Adapted from Bengtsson et al. [20].

## 3. Mechanistic Insights into AAV Toxicity

The relationship between DMD pathophysiology and AAV gene therapy toxicity appears to be complex and bidirectional. Severe toxicities including immune-mediated myocarditis, cardiogenic shock, and thrombotic angiopathy appear more pronounced in patients with advanced disease, particularly in non-ambulatory patients with more severe underlying skeletal and cardiac pathology [9,21].

The underlying DMD pathophysiology creates a hostile inflammatory environment that amplifies AAV toxicity through several mechanisms, including chronic muscle inflammation [22], cardiac vulnerability [16], age, disease severity [9], and dystrophin immunogenicity [22].

### 3.1. Pathophysiology of DMD: Primer

Dystrophin serves as a critical structural linker between cytoskeletal γ-actin and the dystrophin-associated protein complex (DAPC), anchoring the intracellular cytoskeleton to the extracellular matrix. This molecular structure is essential for maintaining sarcolemmal stability during repetitive cycles of muscle contraction and relaxation. In the absence of dystrophin, disruption of the cytoskeleton–extracellular matrix impairs the membrane integrity, making the sarcolemma very susceptible to contraction-induced injury. Increased membrane fragility leads to abnormal permeability to calcium and other small molecules, initiating intracellular signaling cascades that promote myofiber dysfunction, necrosis, and cell death.

Ongoing myofiber necrosis produces a chronic imbalance between muscle degeneration and regeneration. This cycle is characterized by persistent inflammation, upregulation of pro-inflammatory cytokines, and progressive extracellular matrix remodeling. Over time, functional myofibers are progressively replaced by fibrotic and adipose tissue, leading to irreversible muscle weakness and functional decline. Progressive myocardial damage leads to dilated cardiomyopathy [23].

### 3.2. Vector Dose and Biodistribution

Vector dose is the most critical determinant of toxicity risk and varies based on the therapeutic target and the AAV serotype. Systemic administration requires substantially high doses given the larger volume of distribution, and it is typically delivered at total doses of approximately 10^14^–10^16^ vector genomes (vg) per patient, compared with 10^11^–10^13^ vg per patient for localized or targeted delivery approaches. Notably, the recommended dose of delandistrogene moxeparvovec-rokl is 1.33 × 10^14^ vg per kg of body weight for patients weighing less than 70 kg and a fixed dose of 9.31 × 10^15^ vg total for patients weighing 70 kg or greater [24].

The vector doses for systemic gene delivery in DMD are two to three orders of magnitude higher than those typically used for liver-targeted treatments. Recent studies indicate that vector biodistribution is predominantly influenced by the liver, where AAV accumulates and triggers dose-dependent hepatocellular stress [16]. Non-target organ injury has been documented in both animal and human studies at supraphysiological doses (>10^14^ vg/kg). Recent studies also identified lethal immunotoxicity in high-dose systemic AAV therapy, marked by hepatic necrosis, microvascular thrombosis, and a cytokine storm [16,25]. In DMD mouse models, the overexpression of micro-dystrophin in cardiac tissue led to sarcomeric disarray and premature mortality [26,27], suggesting that off-target tropism and expression imbalance may be contributing factors.

### 3.3. Innate Immune Activation and Complement Pathways

In contrast to natural viral infections, which typically begin with low genome copy numbers and expand through replication, systemic administration of AAV results in an immediate, supraphysiologic vector genome load. This acute exposure leads to widespread tissue biodistribution and high intracellular vector copy numbers shortly after infusion. As circulating and intracellular vector genomes decline over time, a subset of episomal vector DNA persists within transduced cells, supporting long-term transgene expression. The initial high vector burden is thought to drive innate immune activation through pattern-recognition receptors sensing viral capsid and vector DNA, as well as adaptive immune responses against AAV capsid and, in some cases, the transgene product. These immune responses have been associated with dose-dependent toxicities, including hepatotoxicity, complement activation, thrombocytopenia, and systemic inflammatory responses [28,29,30].

While early animal studies showed mild inflammatory responses, clinical studies revealed innate immune pathways triggered by AAV vectors have significant clinical implications for immunogenicity. TLR9-mediated recognition of the AAV genome is the primary mechanism. Endosomes of plasmacytoid dendritic cells (pDCs) recognize unmethylated CpG motifs in the vector DNA. Activation of the TLR9>MyD88 pathway activates the type I interferon production and results in CD8+ T cell activation through conditioning of antigen cross-presenting DCs. There are also alternative innate pathways that do not utilize the TLR9 pathway. pDCs can activate the IL-1R1>MyD88 signaling pathway via IL-1 cytokine production. TLR2 recognition of the AAV capsid has also been demonstrated [28].

The complement system is the critical component of the immune response to AAV. Complement activation at high systemic vector doses is a critical mechanism leading to severe clinical toxicities. Both classical and alternative complement pathways are triggered by the AAV capsid. This dual mechanism, especially at high doses (≥1 × 10^14^ vg/kg), contributes to severe toxicities, including thrombotic microangiopathy [30]. The binding of pre-existing anti-AAV immunoglobulin G (IgG) antibodies to AAV capsids triggers the antibody-dependent classical pathway. High neutralizing antibody titers (>1:100) increase the vector uptake and complement activation compared to the lower titers. In seronegative individuals, activation of the complement cascade through the alternative pathway was also demonstrated in recent studies. The markers of the alternative pathway (complement antigens Ba and Bb) were shown to increase in systemic gene therapy-treated patients, confirming the presence of the alternative pathway. Recent studies have also revealed that thrombotic microangiopathy related to AAV gene therapy primarily depends on the antibody-dependent classical pathway but is exacerbated by the alternative pathway. Notably, genetic variability may modulate the potential activation of the alternative pathway [31,32].

The temporal pattern of complement action in the rAAV9-based therapy fordadistrogene movaparvovec trial (NCT03362502) demonstrated C3 and C4 depletion, with elevated C5b-9 (membrane attack complex) occurring approximately 7 days after the infusion. This timing correlated with a rapid decrease in platelet levels and an increase in anti-AAV9 antibodies in patients who developed TMA. Type 1 interferon levels were shown to increase within 2–7 days after systemic infusion, indicating that the early innate immune system activates before the complement pathway activation [21]. Interestingly, both full and empty AAV particles are equally potent in inducing cytokine secretion and complement activation, indicating the capsid itself, not the genome, is the main driver of complement activation [33].

### 3.4. Adaptive Immunity and Cytotoxic T-Cell Responses

Up to 60% of the general population has pre-existing neutralizing antibodies (NAbs) against common AAV serotypes (AAV2, AAV9) [34]. NAbs attach to circulating vectors, forming immune complexes that trigger the activation of complement and antigen presentation. Additionally, AAV capsid peptides displayed on major histocompatibility complex class I (MHC I) molecules trigger cytotoxic T-cell responses targeting transduced cells, as evidenced in liver gene therapy trials [35,36]. Viral capsid proteins, transgene-encoded therapeutic proteins, and epitopes from alternative reading frames within the transgene may activate cytotoxic T lymphocytes [37,38]. The severity of the immune responses varies based on the vector type. Despite low immunogenicity, AAV vectors can also trigger the transgene-specific CD8+ T cell response.

### 3.5. Transgene Overexpression and Proteostatic Stress

Cardiac muscle is very sensitive to transgene overexpression, and expression levels in the cardiac tissue can be 10-fold higher than skeletal muscle following a systemic gene delivery. In DMD animal models, supraphysiologic micro-dystrophin expression in the heart has been associated with myocyte damage, potentially due to interference with sarcolemmal protein assembly and overload of protein quality-control systems. Excessive transgene expression exceeds the cellular protein degradation capacity, activating stress cascades that lead to cardiomyocyte death. Abnormal protein homeostasis and elevated micro-dystrophin expression may disturb the balance of the cardiomyocyte membrane, leading to additional cellular stress [26,39].

### 3.6. Genome Integration and Genotoxicity

The risk of AAV-mediated genotoxicity through genome integration is considered theoretical in humans, as no confirmed cases of mutagenesis have been reported in clinical trials. However, AAV vectors can integrate into host DNA at low frequencies and long-term safety follow-up is recommended [40]. Hepatocellular carcinoma related to insertional mutagenesis has been identified in neonatal mice [41], but this has not yet been observed in humans. This genotoxicity is thought to be related to rapid hepatocyte proliferation during development rather than a generalizable risk [42]. In DMD CRISPR-AAV studies, long-term persistence of edited myofibers was observed, but unintended insertions and large genomic rearrangements were also detected. High levels of AAV integration (up to 47%) into Cas9-induced double-strand breaks (DSBs) are identified [43].

### 3.7. Host and Disease-Specific Susceptibility

Patients with a reduced left ventricular ejection fraction (LVEF) may have a higher risk of myocarditis; therefore, trials have excluded patients with LVEF < 40% based on this concern. Specific DMD mutations may trigger T-cell-mediated immune responses against the transgene. This concern led to the exclusion of specific mutations from micro-dystrophin clinical trials. For example, patients with any deletion of exons 8 and 9 are excluded from treatment with delandistrogene moxeparvovec-rokl. Age has an impact on safety, efficacy, and immune response to AAV gene therapy. For delandistrogene moxeparvovec-rokl, clinical data showed a more favorable safety profile in the younger, ambulatory group than in the older, non-ambulatory group [7]. In the fordadistrogene movaparvovec trial, severe treatment-related events, including fatal cardiogenic shock, were observed in the older, non-ambulatory group [44]. Regarding efficacy, younger patients with less advanced disease showed more robust transgene expression, though functional outcomes showed mixed results [7]. Delivering therapy early in life creates a therapeutic paradox. Continued muscle cell proliferation during growth dilutes transgene expression, limiting durability, necessitating higher doses, which in turn may increase toxicity risk. On the other hand, delaying treatment until later ages may reduce muscle transduction [18]. Beyond age, pre-existing immunity, and genotype, delandistrogene moxeparvovec-rokl is not recommended in patients with pre-existing liver disease, active hepatic infection, recent vaccination, or active/recent infections within weeks [45].

### 3.8. Clinical Manifestations of Toxicity Following Gene Transfer

AAV-mediated gene therapy for neuromuscular diseases is typically administered intravenously (with one notable exception discussed below) and requires high systemic doses, generally ranging from 1 × 10^14^ vg/kg to 2 × 10^14^ vg/kg, to achieve adequate skeletal muscle transduction. Adenoviral vector-induced immune responses have been recognized as a major barrier to gene therapy ever since the tragic death of Jesse Gelsinger in 1999, which was triggered by a severe immune reaction to the adenoviral vector [46]. This event prompted a shift in the field toward AAV vectors; however, AAVs can also elicit immune responses. AAV vector-related adverse events range from common symptoms such as nausea and vomiting to serious, potentially life-threatening complications [25]. Overall, early adverse events are generally attributed to immune responses directed against the AAV capsid [25], while delayed-onset events are more often associated with adaptive immune responses targeting the transgene [47].

#### 3.8.1. Acute Liver Injury

Current AAV vectors exhibit strong hepatic tropism, which can trigger both innate and adaptive immune responses, leading to inflammation and endothelial injury. Clinically, these processes often correlate with elevated liver enzymes on laboratory testing. Liver toxicity has been observed in humans since the early clinical trials. Patients with hemophilia B, who received an AAV2/8 vector delivering factor IX, demonstrated elevations in serum liver transaminases accompanied by a decline in factor IX expression several weeks after vector infusion [48]. On the opposite spectrum from asymptomatic transaminasemia, liver injury can be a serious and life-threatening complication, with the potential to influence the trajectory of an entire gene therapy program. In the clinical trial for X-linked myotubular myopathy (XLMTM, MTM1 gene), several participants experienced severe hepatobiliary complications, including four participant deaths and additional cases of significant but non-fatal liver dysfunction. The liver injury described in these cases presented as progressive, severe cholestatic liver failure, with serious adverse events occurring between 37 and 142 days following AAV administration. Early liver enzyme elevations were typically detected 1 to 4 weeks after treatment. According to available reports, liver histology did not demonstrate immune cell infiltrates, suggesting that the process may not have been immune-mediated [49].

In the DMD field, additional fatalities have also been observed following systemic AAV-mediated gene therapy. Two non-ambulatory adolescent males treated with Sarepta’s AAVrh74 gene therapy platform died of acute liver failure: one individual received the commercially available product Elevidys and the other was treated as part of the ENVISION clinical trial program. In the first case, a recent cytomegalovirus (CMV) infection was believed to have contributed to the severity of liver injury. Following these events, the FDA added a boxed warning for acute liver injury, liver failure, and death and restricted Elevidys to ambulatory boys aged 4 years and older. As of this writing, Sarepta has paused dosing Elevidys in non-ambulatory patients while risk-mitigation strategies are evaluated, including modifying the immunosuppressive protocol that accompanies gene treatment [50].

Based on these and other experiences in the gene therapy field, it is critical that clinicians carefully assess liver function prior to administering gene therapy. This evaluation should include γ-glutamyl transferase (GGT), total and direct bilirubin, and standard transaminases (ALT and AST), with the understanding that ALT and AST are often chronically elevated in DMD patients and may be less specific indicators of acute toxicity. It is recommended that a hepatologist with experience in gene therapy be available at the time of dosing and in the days and weeks following administration, so that any early signs of hepatic dysfunction can be promptly evaluated and managed. Additionally, the safety profile of systemic AAV gene therapy in individuals with pre-existing liver pathology remains unknown, underscoring the need for heightened caution and thorough pre-treatment screening [51].

#### 3.8.2. Thrombotic Microangiopathy

Thrombotic microangiopathy (TMA) has emerged as an uncommon but potentially severe immune-mediated complication of systemic AAV-based gene therapy for DMD, with cases reported across multiple AAV programs, capsid types, and patient populations [52]. TMA following AAV gene therapy typically manifests 7–10 days post-infusion with rapid platelet decline, accompanied by C3 and C4 depletion and elevated C5b-9 levels [21]. The temporal sequence involves peak vector blood concentration at 4–7 days, Type 1 interferon cytokine elevation within 2–7 days, and a particularly rapid rise in neutralizing antibodies and total anti-AAV antibodies in the first 1–2 weeks post-infusion in affected patients. The etiology of TMA following gene transfer includes capsid-related immune responses resulting in activation of both classical and alternative complement pathways. Single-cell analysis has revealed the expansion of T-cell clonotypes post-treatment, with some expanded clumps assignable to prior herpesvirus infections, present in patients who developed TMA [53].

Cases of TMA were initially limited to pediatric DMD patients receiving micro-dystrophin transgenes via AAV9 [21] but have subsequently been reported across a range of disorders, transgenes, promoters, and AAV capsid types. In the fordadistrogene movaparvovec (NCT04281485) phase 1b trial, three of 22 participants experienced clinical TMA [21]. All three patients were hospitalized, received supportive care and anti-complement therapy, and their events resolved. The SGT-001 trial (NCT03368742) was temporarily halted twice due to thrombocytopenia and reduced red blood cell counts attributed to complement activation [18]. In the SMA world, a fatal case of systemic TMA was reported following onasemnogene abeparvovec in a 6-month-old child who carried a potential genetic predisposition in the complement factor I gene [54].

#### 3.8.3. Immune-Mediated Myositis

The pathogenesis of immune-mediated myositis (IMM) is particularly interesting because it occurs only in a subset of patients. IMM appears to develop in individuals who have lost self-tolerance to specific regions encoded by the transgene, leading to an immune response [47]. The first evidence of IMM came from a 2010 study in which six patients with DMD received intramuscular AAV2.5 gene therapy. Three of these patients developed T-cell responses to dystrophin, but no dystrophin-specific antibodies were detected. Notably, two patients had T cells targeting dystrophin prior to gene therapy, suggesting prior priming from revertant fibers [47]. More recently, in the ENDEAVOR study (NCT04626674) using Sarepta’s micro-dystrophin, two cases of IMM were observed in patients with mutations involving exons 3–43 and 8–9, approximately one month after gene therapy. One patient required plasmapheresis and tacrolimus and ultimately recovered, with residual weakness as a sequela [55]. To date, six cases of IMM have been reported following gene therapy with different AAV vectors and various mini- or micro-dystrophin constructs: five patients had large deletions, and one had an exon 8–9 mutation. Mapping these patients’ constructs revealed that all lacked hinge 1, indicating this region as a potential immunogenic target [47,56].

Clinically, IMM leads to severe weakness, often affecting core and facial muscles more than the limbs, along with muscle pain, dysphagia, and dyspnea. Symptom onset typically occurs 3–6 weeks post-infusion [45]. Respiratory weakness can be severe enough to require noninvasive or invasive ventilation. Supporting evidence for IMM includes elevated CK (with or without myoglobinuria), muscle edema on MRI, and T-cell infiltration on muscle biopsy when performed. In many cases, IMM occurs concomitantly with myocarditis. To mitigate these effects, immune-suppression strategies are employed, although the optimal regimen and timing are still under investigation.

#### 3.8.4. Myocarditis and Cardiomyopathy

Micro-dystrophin gene therapy has biological effects on cardiomyocytes that may be both beneficial and adverse. Consequently, understanding the impact of gene therapy on the heart is fundamental. Myocardial injury following systemic AAV administration may arise through several mechanisms, including innate immune activation within cardiomyocytes and vascular endothelium, humoral and cellular immune responses against the AAV capsid, or immune recognition of the newly expressed micro-dystrophin transgene within cardiac tissue. As a result, myocarditis may occur immediately after administration or emerge over the subsequent days to weeks. Myocarditis—ranging from asymptomatic troponin elevation to clinically significant cardiac dysfunction—has been reported 1–2 weeks after vector infusion [57]. For this reason, close cardiac monitoring before and after gene therapy, as well as continuation of immunomodulatory medications for at least 60 days post-infusion, is now recommended [58].

Although presentations vary in severity, the potential for serious outcomes is well documented. A fatal case occurred in a 16-year-old non-ambulatory patient who received a high dose of AAV (3 × 10^14^ vg/kg) in the Phase Ib trial NCT03362502 sponsored by Pfizer. The patient died of cardiogenic shock 6 days post-infusion [59]. Pre-treatment cardiac MRI showed an LVEF of 56% according to the central reader, but 35–45% by the local cardiologist; interpretation was limited by pronounced myocardial fibrosis [44]. Another fatal case was reported in an N-of-1 study, in which a 27-year-old patient with DMD received high-dose systemic rAAV9 carrying a CRISPR-activating transgene. Several days after infusion, he developed mild cardiac dysfunction and a pericardial effusion, rapidly progressing to acute respiratory distress syndrome (ARDS) and subsequent cardiac arrest. Autopsy demonstrated severe diffuse alveolar damage, consistent with a fulminant innate immune-mediated ARDS [16].

These experiences draw attention to critical lessons for the field. Rigorous inclusion and exclusion criteria are essential, particularly regarding cardiac fibrosis and advanced disease stage, as non-ambulatory patients appear to carry a higher risk. Intensive post-treatment surveillance, including serial troponin, brain natriuretic peptide (BNP), inflammatory markers, and cardiac imaging (echocardiography and/or cMRI), should be performed immediately after gene therapy and continued for several months. Long-term follow-up may also be warranted, given the potential delayed or chronic cardiac effects. Importantly, the risk of fatal cardiac events must be clearly communicated during the informed consent process as a real (not merely theoretical) risk. At present, no guidelines or published data address the safety of gene therapy in DMD patients with severe left ventricular dysfunction (LVEF < 40%). This reflects the fact that pivotal studies supporting Elevidys approval included only patients with LVEF ≥ 40%. In the ENDEAVOR study, the lowest baseline LVEF reported was 48.9% [60].

#### 3.8.5. Hemophagocytic Lymphohistiocytosis

Hemophagocytic lymphohistiocytosis (HLH) is a rare but serious hyperinflammatory syndrome characterized by excessive activation of macrophages, T cells, and cytokine release, leading to fever, cytopenias, hepatosplenomegaly, liver dysfunction, coagulopathy, and multi-organ involvement. Although HLH is associated with genetic immunodeficiencies, infections, or malignancies [61], there have been emerging reports of HLH following AAV gene therapies. The first published case arose in a 3-year-old child with SMA treated with onasemnogene abeparvovec who developed fever, rash, hepatosplenomegaly, cytopenias, hyperferritinemia, hypertriglyceridemia, hypofibrinogenemia, and elevated inflammatory markers ~36 h after infusion. The event was resolved after bolus high-dose methylprednisolone followed by tapering [62]. One additional case of HLH was identified in the real-world pharmacovigilance study of onasemnogene abeparvovec, indicating that HLH is a known, albeit rare, risk associated with this gene therapy [63]. More recently, a case was publicly disclosed by Neurogene Inc. in the context of a high-dose AAV trial for Rett syndrome. A pediatric patient treated with a high vector dose (3 × 10^15^ vg total) administered via ICV died approximately two weeks after dosing due to HLH. Following the event, Neurogene implemented a monitoring and treatment algorithm for HLH in its ongoing trial program and recommended early monitoring post-dosing for the hallmark “three F’s” of HLH: fever, elevated ferritin, and falling blood counts (cytopenia). To date, no cases of HLH have been reported following AAV gene therapy in DMD; however, the risk cannot be excluded, particularly with high-dose administration. HLH may also be underrecognized or misattributed to other disease manifestations, such as liver failure, sepsis, or multi-organ dysfunction. Consequently, many emerging DMD trials are now implementing prospective HLH monitoring, including serial ferritin measurements, inflammatory markers, and complete blood counts, in addition to standard liver function surveillance.

#### 3.8.6. Dorsal Root Ganglia Toxicity

Neurotoxicity in the peripheral nervous system (PNS) following AAV administration is difficult to recognize clinically, as it rarely presents with overt neurological signs. Dorsal root ganglia (DRG) involvement was first reported in 2014, when neuronal degeneration, inflammation, and gliosis were observed in the DRG of mice treated intrathecally with AAV9 [64]. In non-human primates, high-dose AAV administration can induce DRG toxicity when delivered intrathecally, intracerebroventricularly (ICV), or intracisternal magna (ICM), and to a lesser extent following intravenous (IV) delivery [65]. Although systemically administered AAV vectors can reach the DRG—particularly at high doses, as the DRG is not protected by the blood–brain barrier—DRG toxicity has not been reported in humans with DMD treated with IV AAV vectors to date. This remains important to monitor, especially as gene therapy trials for Duchenne using intrathecal delivery are being developed (NCT06817382). Clinical experience from other neuromuscular diseases illustrates the potential risk. In SOD1-associated ALS, intrathecal delivery of an AAV vector encoding a microRNA-targeting SOD1 led to the emergence of AAV capsid-specific T cells and neurological symptoms consistent with DRG toxicity on MRI approximately four weeks post-treatment [66]. Another example comes from post-marketing data for onasemnogene abeparvovec (Zolgensma), administered IV, where DRG toxicity is a potential risk; however, interpretation is complicated because DRG degeneration is part of SMA pathophysiology [67].

Detecting DRG toxicity clinically is particularly challenging because it primarily affects the sensory component of the PNS. When present, symptoms may include gait abnormalities, proprioceptive deficits, and reduced sensory nerve conduction amplitudes, which can be especially difficult to detect in younger patients. These considerations highlight the need for close, longitudinal monitoring, including age-appropriate neurological examinations, symptom surveillance, MRI, and nerve conduction studies when feasible, as well as the investigation of emerging soluble biomarkers such as the neurofilament light chain [68].

## 4. Therapeutic and Clinical Implications

AAV-based gene therapy for DMD has demonstrated remarkable promise, but its clinical success depends on the careful management of vector-related toxicity and immune responses. Lessons from both preclinical studies and early clinical trials identified key factors that impact safety, efficacy, and long-term outcomes, modifying strategies for patient selection, dosing, vector design, and immune modulation.

### 4.1. Vector Design Innovations

Recent advances in vector design aim to improve muscle tropism and reduce hepatic uptake. Capsid is a key factor for AAV tissue tropism. Novel capsid variants, including AAVMYO, MyoAAV, AAVLK03, and AAV-ΔVP1, are being engineered to reduce immunogenicity and improve delivery to targeted tissues. RGD-motif-containing myotropic AAV represents a major advancement by achieving 5-to-10-fold enhanced heart and muscle transduction while liver detargeting. These engineered capsids have the arginine–glycine–aspartate (RGD) amino acid motif on the surface that binds to integrin heterodimers on muscle cells [69,70]. These AAVMYO capsids were engineered through semi-rational bioengineering that merges AAV capsid and peptide library screens. Both AAVMYO2 and AAVMYO3 show enhanced tropism for skeletal muscle, diaphragm, and cardiac tissue while simultaneously reducing off-target liver transduction. In a mouse model of X-linked myotubular myopathy, these vectors showed extended survival and improved muscle strength. In the *mdx* mouse model, AAVMYO led to robust transgene expression and improved muscle functional testing [70,71]. In parallel, tissue-specific promoters (e.g., MHCK7) and microRNA-regulated expression cassettes restrict expression to target muscle tissue, minimize off-target expression, and reduce the risk of hepatic toxicity.

Several strategies have been developed to reduce AAV immunogenicity, including CpG depletion, inclusion of TLR9 inhibitory sequences, muscle-specific promoters in combination with miR142-3pT sequences for hematopoietic detargeting, and transgene optimization to exclude immunogenic epitopes. Each strategy has important limitations. For example, CpG depletion substantially reduces CD8+ T-cell responses by eliminating TLR9 ligands that activate plasmacytoid dendritic cells. However, complete CpG elimination from the AAV genome may not always be feasible [72,73]. Incorporating TLR9 inhibitory oligonucleotide sequences into the vector genome may represent an alternative strategy; however, this approach did not entirely prevent an inflammatory response in preclinical studies [74]. A combination of muscle-specific promoters with hematopoietic-specific microRNA target sequences prevents transgene expression in antigen-presenting cells; however, in the setting of dystrophic muscle pathology, this strategy does not entirely prevent an immune response [75]. Modifications in the transgene structure to decrease immunogenic epitopes may entirely affect therapeutic efficacy. For example, Hinge 1 (H1) contains immunogenic epitopes triggering the CD8+ T-cell response. However, the removal of the H1 region in micro-dystrophin products compromises muscle protection [76]. The number of spectrin-like repeats in micro-dystrophin constructs also affects both function and immunogenicity. Constructs with fewer repeats may reduce the antigenic load but can reduce the mechanical flexibility and force transmission, which is critical for muscle fiber protection. The C-terminal domain binds to dystroglycan and is important for sarcolemmal stability. Complete deletion of the CT domain (ΔCT) may reduce immunogenic epitopes but also removes functional binding sites, creating a difficult trade-off between reducing immunogenicity and sacrificing efficacy [74].

### 4.2. Vector Manufacturing

Manufacturing-related toxicity mechanisms may include the presence of empty capsids, residual host cell proteins, and DNA contaminants, which may trigger innate immune responses. Novel manufacturing strategies such as polymer-based coating of AAV particles can improve transduction efficacy and muscle tissue expression [77].

### 4.3. Patient Selection and Timing

Early patient selection and timing are critical for the safety and efficacy of gene therapy. Liver fibrosis negatively impacts AAV-mediated gene transfer to hepatocytes and reduces transduction efficiency [78]. Active inflammation and hepatocellular injury can exacerbate AAV-related liver toxicity; therefore, patients with active hepatic disease and baseline transaminase elevations are exclusion criteria for gene therapy. Hemodynamic alterations in heart failure may impact vector distribution and patients with significant heart failure are excluded from AAV gene therapy. Pre-existing neutralizing antibodies against the AAV capsid are also a major barrier to gene therapy eligibility. Screening for baseline complement activity and genetic predisposition to complement dysregulation may help identify patients who are at high risk for AAV toxicity [79]. Certain genetic mutations increase the risk of immune-mediated myositis in gene therapy. For example, patients with deletions of exons 8 and 9 were excluded for delandistrogene moxeparvovec-rokl.

Another critical consideration is the risk associated with dosing older and/or non-ambulatory patients. This risk is multifactorial and largely driven by advanced disease progression. Non-ambulatory patients have markedly reduced lean muscle mass, which can significantly alter AAV vector biodistribution [80]. In this setting, a greater proportion of the vector may be diverted away from skeletal muscle toward off-target organs such as the liver or lungs, potentially increasing the risk of severe toxicities. In addition, older and non-ambulatory patients frequently have advanced cardiac involvement. A reduced left ventricular ejection fraction (LVEF) is a known risk factor for acute myocarditis and other serious cardiac complications, which may be exacerbated in the context of systemic gene transfer. Similarly, older patients often have restrictive lung disease, further increasing vulnerability to pulmonary complications. These factors highlight the need for enhanced patient selection strategies that extend beyond ambulatory status alone. Multidimensional assessments should be incorporated, including quantitative measures of cardiac function, comprehensive pulmonary testing, and, where feasible, body composition analysis, alongside an integrated evaluation of overall disease stage. Equally important is a patient-centered approach to risk–benefit assessment and a transparent discussion with patients and families that explicitly addresses individual vulnerabilities and clearly outlines potential risks (including risk of death) versus expected benefits to support informed and shared decision-making.

### 4.4. Immune Modulation

Immune modulation remains a cornerstone of safety, with corticosteroids as frontline prophylaxis for transaminitis and gene therapy-related toxicities. Whether administered as daily or weekend dosing, glucocorticoids—including prednisone/prednisolone, deflazacort, and more recently, vamorolone—are the standard of care for DMD and are associated with a reduced risk of losing clinically meaningful motor function over time [81]. The mechanisms of action of corticosteroids are multifaceted. Beyond broad anti-inflammatory effects within muscle tissue, corticosteroids modulate innate immune responses by acting on pattern-recognition receptor signaling pathways, reducing plasma cell differentiation, and influencing complement activation—all of which may be beneficial in the context of gene therapy. Importantly, chronic corticosteroid use in DMD is also associated with reduced pre-existing anti-dystrophin T-cell immunity: approximately 8% of steroid-treated DMD patients demonstrate dystrophin-specific T-cell responses, compared with substantially higher rates in steroid-naïve patients; and deflazacort appears more effective than prednisone in suppressing these immune responses [82].

A unique challenge in DMD gene therapy is that most patients are already receiving baseline corticosteroids at the time of treatment. Current gene therapy protocols typically recommend adding prednisone at 1 mg/kg/day (up to 60 mg daily) on top of baseline steroid therapy [83]. This cumulative exposure raises several concerns. Beyond amplifying the well-known adverse effects of chronic corticosteroid use, increased steroid burden may confound safety assessments. For example, corticosteroid-induced hepatic steatosis may complicate the interpretation of post-gene therapy hepatotoxicity and transaminase elevations. In addition, corticosteroids significantly alter body composition, increasing adiposity and reducing lean muscle mass. This raises important questions regarding AAV dosing strategies, which are currently weight-based and may not accurately reflect the biologically relevant target tissue. As a result, there is growing interest in dosing based on lean body mass rather than total body weight [84].

Looking forward, future studies may benefit from stratifying outcomes based on baseline corticosteroid exposure. This approach may become increasingly feasible as clinical trials expand enrollment to younger patients to demonstrate safety and efficacy in steroid-naïve or minimally exposed patients, allowing clearer dissection of the immunologic and safety interactions between corticosteroids and gene therapy.

Although this is outside the scope of the present paper, it is worth noting that additional immune-suppressive strategies that can either precede or follow gene transfer or be used as a rescue regimen (depending on the clinical trial) include the mTOR inhibitor sirolimus (rapamycin) and the nucleotide synthesis inhibitor mycophenolate mofetil; T-cell-targeted calcineurin inhibitors such as tacrolimus and cyclosporine; B-cell-directed anti-CD20 therapy (rituximab); complement inhibitors including the C3 inhibitor pegcetacoplan and the C5 inhibitor eculizumab; and cytokine-targeted therapies such as the IL-6 receptor inhibitor tocilizumab and the IL-1 receptor antagonist anakinra [31,85,86,87]. Comprehensive studies are needed to establish the optimal protocols for immunomodulation across different AAV serotypes, routes, and patient populations [88].

### 4.5. Clinical Monitoring and Risk Mitigation

Comprehensive clinical monitoring is essential to maximize safety and detect adverse events early in gene therapy. Baseline assessments should include liver panels, cardiac biomarkers, complement function, AAV serotype antibodies, as well as an electrocardiogram and echocardiogram. Following infusion, patients should be closely monitored for 6–12 h for signs of fever, hypotension, or thrombocytopenia. Daily or every other day monitoring during the first week should include ALT/AST/GGT, bilirubin, coagulation panel, fibrinogen, troponin, complete blood counts, complement components (C3a/C5a), BNP, creatine kinase, urinalysis, and as-needed ferritin. Long-term follow-up can include MRI for hepatic steatosis or fibrosis and annual cardiac MRI (Table 2).

## 5. Regulatory and Ethical Considerations

Regulatory agencies have begun re-evaluating systemic AAV dosing thresholds following reported deaths in DMD clinical trials and during post-approval commercial use [15]. Risk-mitigation frameworks now require independent safety review boards, mandatory reporting of complement activation, and enhanced informed-consent language emphasizing potential fatality. Post-marketing surveillance is essential because patient heterogeneity (genetic background, comorbidities, steroids, infections) can reveal safety risks not observed in tightly controlled clinical trials.

## 6. Future Perspectives

Many limitations affect current AAV delivery methods for DMD. As described above, these include activation of the patient’s immune system and the fact that, after intravenous administration, the gene therapy product cannot be re-dosed. To overcome some of these challenges, a newer strategy has been explored: intrathecal administration, typically performed via lumbar puncture. This delivery route offers the additional advantage of enabling more effective crossing of the blood–brain barrier, which is particularly important given that at least one-third of patients with DMD exhibit neuropsychiatric and cognitive manifestations. These range from autism spectrum features to reduced IQ and, in some cases, significant developmental delay [89]. An ongoing Phase Ib study (NCT06817382) is evaluating the safety of a single intrathecal injection of an AAV9-delivered micro-dystrophin gene therapy, administered at a fixed (non-weight-based) dose, in ambulatory DMD patients younger than 5 years. The first patient was dosed in July 2025. Although still in early trials, it is not yet clear how intrathecal therapy compares in efficacy to the intravenous form. It can however be administered at lower doses, which may result in a more favorable immune and safety profile. Intrathecal delivery also allows some CNS penetration, potentially addressing cognitive and behavioral symptoms of DMD that have historically received less attention. Additionally, this approach might be feasible in patients with pre-existing anti-AAV antibodies, potentially expanding eligibility to individuals who were previously excluded from gene therapy.

## 7. Conclusions

The field of AAV-based gene therapy for DMD is rapidly evolving with tremendous research in the field. Recent tragic outcomes related to AAV toxicity have continued to shape the field, highlighting the urgent need for more AAV biology-informed and patient-centered considerations in DMD. Since most AAV-associated toxicities are dose-dependent, more potent therapeutic designs may hold promise for decreasing the AAV vector dose. In combination with better-designed capsids, transgene cassettes with targeted delivery strategies, along with an optimized immune modulation, will allow safer and more effective AAV-based therapies in DMD. A comprehensive approach integrating institutional readiness, optimized monitoring, transparent safety reporting, timely intervention, and equitable access are key considerations to improve outcomes for AAV-based therapies. Achieving this goal depends on close collaboration among basic scientists, clinicians, and regulatory agencies to advance gene therapy safely and effectively.

## 8. Patents

L.V.G holds a Patent Application related to the AAV.U7snRNA-mediated therapy to skip exon 17 in the *DMD* gene.

## Figures and Tables

**Table 2:** Clinical monitoring of patients following AAV administration.

Type of Immunity	Trigger	Clinical Manifestation	Timing	Mitigation
Pre-existing humoral	Anti-AAV Nabs	Loss of efficacy, complement activation	Immediate	Serotype screening, plasmapheresis
Innate (TLR9/complement)	CpG motifs, immune complexes	Fever, thrombocytopenia, hepatotoxicity	Hours–days	Corticosteroids, complement inhibitors
Adaptive cellular	Capsid peptides on MHC I	Myofiber loss, elevated CK	1–4 weeks	Immunosuppression, vector re-design

## Data Availability

No new data were created or analyzed in this study. Data sharing is not applicable to this article.

## Abbreviations

The following abbreviations are used in this manuscript:

AAV	Adeno-associated virus
ALT	Alanine transaminase
ARDS	Acute respiratory distress syndrome
AST	Aspartate aminotransferase
BNP	Brain natriuretic peptide
bp	Base pair
cDNA	Complementary DNA
CK	Creatine kinase
CMV	Cytomegalovirus
DMD	Duchenne muscular dystrophy
ER	Endoplasmic reticulum
GGT	γ-glutamyl transferase
HLH	Hemophagocytic lymphohistiocytosis
IgG	Immunoglobulin G
IMM	Immune-mediated myositis
IT	Intrathecal
ITR	Inverted terminal repeat
IV	Intravenous
kg	Kilograms
LVEF	Left ventricular ejection fraction
MHC I	Major histocompatibility complex class I
mg	Milligrams
mRNA	Messenger RNA
NAb	Neutralizing antibodies neutralizing antibody
NHP	Non-human primate
NSAA	North Star Ambulatory Assessment
ORF	Open reading frame
pDCs	Plasmacytoid dendritic cells
RNA	Ribonucleic acid
PNS	Peripheral nervous system
SMA	Spinal muscular atrophy
TMA	Thrombotic microangiopathy
vg	Vector genomes
Zolgensma	Onasemnogene abeparvovec
